# Drug-Resistant Tuberculosis in Zhejiang Province, China, 1999–2008

**DOI:** 10.3201/eid1803.110760

**Published:** 2012-03

**Authors:** Xiaomeng Wang, Qian Fu, Zhijun Li, Songhua Chen, Zhengwei Liu, Hugh Nelson, Qun Yang, Zhongwei Jia, Christopher Dye

**Affiliations:** Center for TB Control in Zhejiang, Hangzhou, People’s Republic of China (X. Wang, S. Chen, Z. Liu);; Capital Medical University, Beijing, People’s Republic of China (Q. Fu, Q. Yang);; National Research Institute of Food and Fermentation Industries, Beijing (Z. Li);; International SOS Clinic, Shekou, People’s Republic of China (H. Nelson);; Peking University, Beijing (Z. Jia);; World Health Organization, Geneva, Switzerland (C. Dye)

**Keywords:** isoniazid, rifampicin, multidrug-resistant tuberculosis, MDR TB, tuberculosis and other mycobacteria, bacteria, cross-sectional survey, prevalence, China

## Abstract

To evaluate levels and trends in drug-resistant tuberculosis (TB) in Zhejiang Province, China, we conducted 1 survey in each of 3 years (1999, 2004, and 2008). We found that <5% of new cases were multidrug-resistant TB. The prevalence of multidrug-resistant TB has not increased in new or re-treated cases in this province.

In 2009, China reported results of a nationwide drug resistance survey, which found that 5.7% of new cases of tuberculosis (TB) and 25.6% of re-treated cases were infections with multidrug-resistant TB (MDR TB), i.e., resistance to isoniazid and rifampin (*1*). These results indicated that in 2008 in China, MDR TB developed in ≈100,000 persons, which is ≈25% of the total number of TB cases (440,000) and similar to that in India (*1*).

In China, in addition to the 2008 national survey of TB drug resistance and 10 annual national TB surveys, surveys of TB drug resistance have been conducted in several provinces (*2*–*4*). Zhejiang is one of the few provinces that have conducted a series of cross-sectional surveys from which we can evaluate the scale of the drug-resistance problem at one time point and changes over time.

Data from a sequence of surveys are vital in assessing evolution of resistance to TB drugs in China and ultimately in evaluating the effect of control measures. We report findings of 3 cross-sectional surveys, 1 each of which conducted in Zhejiang in 1999, 2004, and 2008. These surveys included prevalence of MDR TB among TB cases diagnosed in clinics, trends, and risk factors for resistance to isoniazid and rifampin singly and in combination.

## The Study

We would need 784 cases (i.e., 1.96^2^_0.05_0.5(1 − 0.5)/(0.07/2)^2^ = 784) to achieve 95% precision and a margin of error of 7%, and assume no prior knowledge of prevalence of drug resistance, to measure prevalence of any form of drug resistance across the entire province (i.e., not enabling stratification) in each year. Assuming that >10% of samples would be lost, we sought to obtain 900 cases. With no prior information for prevalence of drug resistance at different sites, we randomly selected 30 TB treatment centers in 30 counties (among 90 centers in Zhejiang Province) and anticipated that each center would recruit >30 sputum smear–positive patients. Three surveys were conducted at the same 30 sites to obtain the same sample size in each year (*5*).

Sputum was collected from persons with suspected TB who came to clinics for a diagnosis. Three sputum samples were obtained (morning, midday, and evening), and patients with >2 samples with positive sputum smear results were enrolled in the study. Drug sensitivity tests were performed in provincial reference laboratories by using the percentage method, and results were compared with results for standard drug-resistant strains (*6*). Quality of provincial reference laboratories was ensured by the Republic of Korea Supranational Reference Laboratory (Seoul, South Korea) during 3 surveys, and was evaluated annually by the national reference laboratory in China. Recruitment of consecutive case-patients continued until >30 (often more) were enrolled at each site. Each case-patient completed a questionnaire on medical and medication history.

New cases, re-treatment cases, and cases of MDR TB were defined as described by the World Health Organization (*5*). Prevalence of resistance to isoniazid and rifampin or MDR TB was defined as the number of resistant cases in patients who were given a diagnosis of TB in clinics and tested for drug resistance.

Although surveys were not designed a priori to evaluate time trends for prevalence of drug resistance, we investigated trends by using repeated measures analysis of variance and making appropriately cautious conclusions. Logistic regression models were used to investigate factors associated with single drug resistance and MDR TB. Statistical analysis was performed by using SPSS version 17.0 (SPSS, Inc., Chicago, IL, USA).

Totals of 1,013, 984, and 938 MDR TB case-patients were recruited from routinely diagnosed new and re-treatment case-patients in 1999, 2004, and 2008, respectively (Table 1). In the 3 surveys, 71%, 74%, and 69% of cases were in men, and 17%, 16%, and 10% were re-treatment cases.

**Table 1:** Resistance to 2 TB drugs, by patient age, sex, and treatment status in Zhejiang Province, China, 1999, 2004, and 2008*

Year, group	No. (%) patients			
	Total	Isoniazid	Rifampin	MDR
1999				
Total	1,013	138 (13.6)	116 (11.5)	87 (8.6)
Patients 0–14 years of age	12 (1.2)	1 (8.3)	1 (8.3)	1 (8.3)
Patients 15–64 year of age	808 (79.8)	120 (14.9)	97 (12.0)	78 (9.7)
Patients >65 years of age	193 (19.1)	17 (8.8)	17 (8.8)	8 (4.2)
Male patients	723 (71.4)	100 (13.8)	81 (11.2)	60 (8.3)
Female patients	290 (28.6)	38 (13.1)	35 (12.1)	27 (9.3)
New cases	841 (83.0)	72 (8.6)	51 (6.1)	35 (4.2)
Re-treatment cases	172 (16.9)	66 (38.4)	65 (37.8)	52 (30.2)
2004				
Total	984	159 (16.2)	94 (9.6)	75 (7.6)
Patients 0–14 years of age	3 (0.3)	1 (33.3)	1 (33.3)	1 (33.3)
Patients 5–64 years of age	764 (77.6)	125 (16.4)	74 (9.7)	58 (7.6)
Patients >65 years of age	217 (22.1)	33 (15.2)	19 (8.8)	16 (7.4)
Male patients	730 (74.2)	125 (17.1)	73 (10.0)	61 (8.4)
Female patients	254 (25.8)	34 (13.4)	21 (8.3)	14 (5.5)
New cases	831 (84.5)	102 (12.3)	41 (4.9)	30 (3.6)
Re-treatment cases	153 (15.6)	57 (37.3)	53 (34.6)	45 (29.4)
2008				
Total	938	125 (13.3)	75 (8.0)	56 (6.0)
Patients 0–14 years of age	5 (0.5)	0	1 (20.0)	0
Patients 15–64 years of age	756 (80.6)	103 (13.6)	63 (8.3)	48 (6.4)
Patients >65 years of age	177 (18.9)	22 (12.4)	11 (6.2)	8 (4.5)
Male patients	646 (68.9)	91 (14.1)	55 (8.5)	40 (6.2)
Female patients	292 (31.1)	34 (11.6)	20 (6.9)	16 (5.5)
New cases	842 (89.8)	88 (10.5)	43 (5.1)	28 (3.3)
Re-treatment cases	96 (10.2)	37 (38.5)	32 (33.3)	28 (29.2)

*TB, tuberculosis; MDR, multidrug resistant.

In the 3 surveys, average prevalence of new cases resistant to isoniazid and rifampin and having MDR TB was 10.5% (95% CI 8.4%–12.5%), 5.1% (95% CI 3.6%–6.6%), and 3.3% (95% CI 2.1%–4.5%), respectively. Equivalent percentages among re-treatment cases were 38.5% (95% CI, 28.8%–48.2%), 33.3% (95% CI 23.9%–42.7%), and 29.2% (95% CI 20.1%–38.3%), respectively.

Compared with new cases, re-treatment cases were more likely to be resistant to isoniazid (odds ratio [OR] 1.8, 95% CI 1.2–2.7) and rifampin (OR 6.3, 95% CI 4.2–9.5) or to have MDR TB (OR 9.0, 95% CI 6.4–12.7) (Table 2). Resistance to isoniazid was strongly associated with resistance to rifampin and vice versa (models 1 and 2; OR 19.9, 95% CI 13–31) (Table 2).

**Table 2:** Factors associated with resistance to tuberculosis drugs, Zhejiang Province, China, 1999–2008*

Characteristic	Coefficient	OR (95% CI)	p value
Model 1: Risk factors associated with resistance to isoniazid			
Constant	−4.10	0.02	
Year	0.23	1.26 (0.87–1.84)	0.221
Rifampin	2.99	19.91 (12.91–30.70)	<0.001
Age 0–14 years	0.89	2.43 (0.20–34.90)	0.51
Age 15–64 years	−0.49	0.61 (0.40–0.94)	<0.05
Sex	0.16	1.17 (0.80–1.71)	0.43
Re-treatment	0.62	1.85 (1.24–2.76)	<0.005
Model 2: Risk factors associated with resistance to rifampin			
Constant	−3.33	0.10	<0.001
Year	−0.53	0.59 (0.38–0.93)	<0.05
Isoniazid	2.99	19.85 (12.92–30.51)	<0.001
Age 0–14 year	−1.74	0.18 (0.02–1.29)	0.09
Age 15–64 year	−0.05	1.02 (0.62–1.66)	0.95
Sex	−0.30	0.73 (0.47–1.16)	0.19
Re-treatment	1.84	6.29 (4.15–9.53)	<0.001
Model 3: Risk factors associated with MDR TB			
Constant	−2.78	0.62	<0.001
Year	−0.16	0.86 (0.61–1.21)	0.384
Age 0–14 year	−0.69	0.52 (0.06–4.36)	0.56
Age 15–64 year	−0.45	0.62 (0.40–0.96)	<0.05
Sex	−0.17	0.86 (0.58–1.23)	0.37
Re-treatment	2.20	9.01 (6.39–12.68)	<0.001

*Reference groups are >65 years for age, female for sex, and new for re-treatment. OR, odds ratio; MDR TB, multidrug-resistant tuberculosis.

Prevalence of resistance to isoniazid and MDR TB tended to be lower in case-patients 15–64 years of age than in those >65 years of age (models 1 and 3), but this effect was not shown for rifampin (model 2) (Table 2). There was no significant difference in prevalence of resistance between male and female case-patients across all surveys (models 1–3) (Table 2).

Prevalence of isoniazid and rifampin resistance and MDR TB changed little across the 3 surveys among new and re-treatment cases (Table 1). Time trends for isoniazid prevalence (increase) and MDR TB (decrease) among new cases were marginally significant (*F* = 3.33, p<0.05, and *F* = 1.13, p<0.05) but in opposite directions. There were no significant trends in resistance among re-treatment cases or among men or women.

## Conclusions

Approximately 25% of the world’s MDR TB cases are in China, and it is vital to know whether this number is increasing, decreasing, or stable. There are few data with which to judge trends in drug resistance in China, although a few regions, including Shanghai municipality (*7*), Shenzhen Province (Z. Jia, Y. Yong, unpub. data), and Zhejiang Province (this study), have conducted cross-sectional surveys.

The principal finding of our study is that although drug-resistant TB needs careful management in Zhejiang Province (6% of all TB cases in 2008 were MDR TB and resistance to second-line drugs has also been found in the province; X. Wang, unpub. data), prevalence of isoniazid and rifampin resistance and MDR TB monitored at the same 30 sites changed little during 1999–2008. Although surveys were not designed to detect time trends in drug resistance, prevalence of MDR TB decreased from 8.6% in 1999 to 6.0% in 2008. This decrease in Zhejiang was consistent with changes observed during 2000–2010 in 2 national TB prevalence surveys (*3*,*4*).

Our results contrast with those that MDR TB prevalence increased in nearby Shanghai during 2000–2006. Shen et al. (*7*) reported that introduction of directly observed treatment, short course, and other improved management practices contained spread of drug resistance in Shanghai after 2004, and introduction of similar practices in Zhejiang may also have stopped the increase in MDR TB after 2002. However, the role of improved TB control practices cannot be shown from these data. Nevertheless, possible differences among different sites underline the need for monitoring resistance trends locally and nationally in China. It is also necessary to monitor treatment outcomes, which will be linked to development of drug resistance. In this context, the percentage of patients who sought re-treatment was lower in 2008 than in previous years, which suggested that case management had improved.

The greatest risk factor for resistance to either isoniazid or rifampin in this study was resistance to the other drug, a finding that indicates the high risk for acquiring MDR TB after treatment failure. In this context, and consistent with previous studies (*8*,*9*), prevalence of MDR TB was higher among re-treatment cases than new cases. These results also underscore the need for following good management practices as described by the World Health Organization (*10*).
